# Biomass: A Renewable Source of Fuels, Chemicals and Carbon Materials

**DOI:** 10.3390/molecules25215217

**Published:** 2020-11-09

**Authors:** Juan Carlos Serrano-Ruiz

**Affiliations:** Materials and Sustainability Group, Department of Engineering, Universidad Loyola Andalucía, Avda. de las Universidades s/n, 41704 Dos Hermanas, Seville, Spain; jcserrano@uloyola.es; Tel.: +34-955-641-600 (ext. 2579)

Fossil fuels have been long used as a source of carbon for synthetizing the fuels, chemicals, and carbon-based materials we use on a daily basis. However, as we all know, the massive utilization of fossil fuels is associated with a number of economic, political, and environmental issues, which is pushing governments to search for renewable alternatives. Biomass (i.e., any organic material that comes from plants and animals), being the only source of renewable carbon available on our planet, is the natural replacement for fossil fuels for the generation of these products. Unlike fossil fuel feeds, biomass feedstocks (e.g., starches, sugars, lipids, and lignocelluloses) are highly oxygenated, very reactive, and chemically complex. As a result, new processes, reaction conditions, and catalytic materials will need to be developed in order to accomplish this transition. This tremendous effort is currently being led both by academia and industry, which are working in parallel to develop, improve, and finally scale some of these technologies to the industrial level. The journal *Molecules* has witnessed this effort by publishing within the last two years a significant number of excellent contributions in the field of biomass conversion and valorization. The present Editorial is aimed at summarizing these hot papers, providing the readers with a vision of their relevance and potential to provide groundbreaking technologies in the near future.

The production of chemicals from fossil fuels is a rather complex process that requires a number of functionalization steps to activate the hydrocarbons. In the case of biomass, the high degree of functionalities of the biomass feeds represents an important advantage for simplifying the process, since many of these functionalities are already present in the raw material. A good example of this is provided by Gómez-López et al. [1]. These authors developed a new and simple catalytic process for the production of vanillin, an important chemical for the pharmaceutical, cosmetic, and food industries. The process starts with trans-ferulic acid, which is an important component of lignin (one of the three fractions of lignocellulose). Heterogeneous Cu catalysts supported on TiO_2_ allowed carrying out the oxidation of trans-ferulic acid to vanillin with yields as high as 70%, although additional work is required to improve the stability of the catalyst (i.e., Cu leaching was reported). Pfersich et al. [2] used waste sugar beets as a source of chemicals. The spent sugar beets were hydrothermally treated at mild temperatures, resulting in a liquid phase enriched in 5-hydroxymethylfurfural (HMF). HMF is currently used to produce industrial solvents and renewable polymers. Along with the HMF-enriched liquid phase, the hydrothermal treatment also generated a biochar with a heating value (22.7 MJ/kg) comparable to that of fossil coal samples, which provides additional value to the process.

The impact of polymers on today’s science and technology is huge. Biomass provides the opportunity to generate these important materials from renewable sources. An interesting piece of work on this matter was published by Sag et al. [3]. These authors summarized the most relevant technologies available today for producing bio-based polyesters and epoxy resins for flame retardant purposes. Among the different applications of biopolymers, Castro-Muñoz et al. [4] focused on membranes and, more particularly, on membranes for pervaporation purposes. This review described in detail the separation performance of membranes synthetized from important biopolymers such as sodium alginate, polylactic acid, and chitosan. Chitosan, a biopolymer found in the shells of shrimps and other crustaceans, was chemically modified with Lewis basic groups by grafting thiocarbamate functionalities to the NH_2_ groups of the naturally occurring polymer. The resulting material showed excellent performance in the removal of toxic lead and copper ions from water [5]. Porous chitosan and chitosan–graphene oxide materials were used to prepare Pd catalysts with a very low metal particle size (ca. 1.7 nm) [6]. These catalysts showed excellent performance for the release of hydrogen via the decomposition of ammonium formate at mild temperatures (60 °C). This technology is relevant in that it can be used for on-board hydrogen generation, given the high purity of the gas obtained (i.e., no CO was produced during the decomposition). Deactivation of the Pd catalysts was reported, which was ascribed to Pd sintering.

Biomass can be also used to produce fuels for the transportation sector of our society, which accounts for one third of the total energy consumed in the world. Two renewable transportation fuels have received much attention in recent years, namely, bioethanol and biodiesel. Bioethanol is currently produced by the simple fermentation of biomass sugars. While the fermentation technology is mature and well developed, the large-scale production of bioethanol requires large land extensions and the significant utilization of edible biomass (e.g., corn and sugar cane), from which has arisen some moral issues. This availability issue can be overcome by providing new pathways to ethanol from alternative sources such as biogas-derived ethane, as recently reported by Oh et al. [7]. Biodiesel (i.e., long-chain alkyl esters) is typically produced by the transesterification of triglycerides found in vegetable oils with methanol and homogenous basic catalysts (e.g., NaOH). The utilization of heterogeneous basic catalysts provides an alternative for cleaner and more sustainable biodiesel production, as reported by Botti et al. [8]. Valeric biofuels (esters of valeric acid, which can be obtained from biomass-derived levulinic acid) were reported to be obtained via esterification over lipases immobilized by a sol-gel method [9]. This paper is interesting in that it combines the high regioselectivity of enzymes with the advantages of heterogeneous catalysts (e.g., reusability). Liquid hydrocarbon fuels chemically identical to those currently used in our transportation system can also be produced from biomass. The stringent energy-density and cold-flow requirements of jet fuels prevent oxygenated fuels such as bioethanol and biodiesel from being used in these applications. Conventional jet fuels contain a mixture of hydrocarbons, with a certain chain length (C_9_–C_16_) and chemical structure. The review of Díaz-Pérez et al. [10] summarizes the most important catalytic routes available today for producing these hydrocarbons from biomass. The paper analyzes the different routes from four different viewpoints: the level of maturity, the economics, the CO_2_ emissions, and the complexity of the different processes.

Finally, biomass can be used to prepare carbon materials with wide and relevant applications. For example, soybean residues were used to prepare nitrogen-and-oxygen-co-doped porous carbons with a high surface area (ca. 2000 m^2^/g) and proper porosity (wide micro-/mesopore size distribution) to be used as supercapacitors [11]. The high specific surface area and proper pore-size distribution along with the presence of nitrogen functional groups favored the formation of the electrical double-layer, resulting in materials with outstanding electrochemical performance.
